# Impact of modified dexamethasone administration sequence on infusion reaction incidence in HER2-positive breast cancer: a randomized multicenter trial

**DOI:** 10.1007/s12282-025-01752-0

**Published:** 2025-08-02

**Authors:** Ryoichi Matsunuma, Shigeru Nakagaki, Eiji Nakatani, Masayuki Kikuchi, Noriaki Wada, Kei Yonezawa, Tadahiro Isono, Ryosuke Hayami, Mayumi Kaga, Michiko Tsuneizumi

**Affiliations:** 1 Department of Breast Surgery, Shizuoka Prefectural Hospital Organization, Shizuoka General Hospital, 4-27-1 Kita-Ando Aoi-ku, Shizuoka, 420-8527 Japan; 2 Department of Pharmacy, Shizuoka Prefectural Hospital Organization, Shizuoka General Hospital, Shizuoka, 420-8527 Japan; 3 Department of Biostatistics and Health Data Science, Graduate School of Medical Science, Nagoya City University, Aichi, 466-8550 Japan; 4 Department of Surgery, Japanese Red Cross Shizuoka Hospital, Shizuoka, 420-0853 Japan; 5 Department of Clinical Oncology, Tokyo Dental College Ichikawa General Hospital, Ichikawa, Chiba 272-8513 Japan; 6 Department of Surgery, Shizuoka City Hospital, Shizuoka, 420-8527 Japan; 7 Department of Surgery, Shimada General Medical Center, Shizuoka, 427-8502 Japan; 8 Department of Pharmacy, Tokyo Dental College Ichikawa General Hospital, Ichikawa, Chiba 272-8513 Japan

**Keywords:** Pertuzumab, Trastuzumab, Infusion reaction, Premedication

## Abstract

**Background:**

Infusion reactions (IRs) are common adverse events associated with HER2-targeted monoclonal antibodies, such as trastuzumab and pertuzumab. Although dexamethasone is routinely administered before docetaxel to prevent hypersensitivity, its optimal timing relative to HER2-targeted agents has not been established. This study assessed whether premedication with dexamethasone reduces the incidence of IRs associated with HER2-targeted therapy.

**Methods:**

In this randomized, multicenter trial, 100 patients with HER2-positive early breast cancer were randomized to receive dexamethasone either before (experimental group) or after (control group) HER2-targeted agents. All patients received trastuzumab, pertuzumab, and docetaxel. The primary endpoint was the incidence of IRs during the first treatment cycle. Secondary endpoints included the incidence of grade ≥ 3 IRs, IRs in cycle 2, and overall adverse events.

**Results:**

Incidence of IRs in cycle 1 was significantly lower in the experimental group (24.0%) than in the control group (60.0%) (*P* < 0.001), corresponding to an absolute risk reduction of 36.0%. No grade ≥ 3 IRs occurred in either group. The incidence of IRs during cycle 2 was low and similar between groups (8.0% vs. 10.2%; *P* = 0.703). The incidence of treatment-related adverse events was similar between groups (98.0% vs. 100.0%, *P* > 0.999). Time-course analysis revealed that most of IRs in the control group occurred before dexamethasone administration.

**Conclusions:**

Premedication with dexamethasone before HER2-targeted therapy substantially reduced IRs without additional toxicity. This straightforward, cost-effective modification to the premedication protocol may improve tolerability in HER2-positive breast cancer and other antibody-based therapies.

**Trial registration:**

UMIN000045181 (registered on August 18, 2021).

## Introduction

Dual HER2 blockade with trastuzumab and pertuzumab has become the cornerstone of treatment for HER2-positive breast cancer across various clinical settings, including neoadjuvant, adjuvant, and metastatic therapy [1–6]. The combination of trastuzumab, pertuzumab, and docetaxel has demonstrated significant efficacy, as evidenced by pivotal clinical trials including NeoSphere, CLEOPATRA, and APHINITY [4–6].

Trastuzumab and pertuzumab have complementary mechanisms of action, and they act by targeting distinct domains of the HER2 receptor. Trastuzumab binds to subdomain IV of HER2, inhibiting cleavage and ligand-independent signaling, whereas pertuzumab binds to the dimerization domain, preventing HER2 dimerization [7, 8]. Both agents activate antibody-dependent cellular cytotoxicity, thereby enhancing their antitumor activity [9, 10]. Despite their favorable efficacy and tolerability profiles, infusion reactions (IRs) remain a significant concern, occurring in 13%–40% of patients during initial treatment cycles without premedication [2, 3, 11]. These IRs, characterized by symptoms such as chills, fever, and nausea, can adversely affect quality of life, increase patient anxiety, prolong hospitalization, and compromise treatment adherence.

The current strategies to counter IRs include premedication with antipyretics and corticosteroids. In this setting, dexamethasone is routinely administered before docetaxel to reduce the risk of edema, nausea, and allergic reactions. However, the recent retrospective studies suggest that administering dexamethasone before HER2-targeted therapies may significantly reduce the incidence of IRs as compared to the conventional sequence [3, 12]. This hypothesis is consistent with guidelines recommending corticosteroid premedication as an effective strategy to prevent IRs [3].

This randomized clinical trial was designed to evaluate whether modifying the sequence of dexamethasone administration within the trastuzumab, pertuzumab, and docetaxel regimen could reduce the incidence and severity of IRs in patients with early-stage HER2-positive breast cancer. Specifically, the study compared a modified protocol in which corticosteroids were administered prior to HER2-targeted therapy against the conventional sequence, to determine whether the timing of steroid premedication influences the risk of IRs.

Although the focus of this trial was on HER2-positive breast cancer, the findings may have broader relevance. In various monoclonal antibody therapies where corticosteroid premedication is not routinely implemented, yet IRs remain a concern—such as the initial administration of certain HER2-targeted regimens or monoclonal antibodies used in other solid tumors—optimizing the timing of premedication may represent a simple and generalizable strategy to improve treatment tolerability and safety.

## Patients and methods

### Study design

This randomized, multicenter clinical trial evaluated whether modifying the sequence of dexamethasone administration in combination therapy with trastuzumab, pertuzumab, and docetaxel reduces the incidence of IRs in patients with HER2-positive breast cancer. Patients were randomized in a 1:1 ratio to the control group (conventional premedication sequence) or the intervention group (dexamethasone before HER2-targeted therapies). Randomization was stratified by treatment setting (neoadjuvant or adjuvant). This study is registered with the University Hospital Medical Information Network, number UMIN000045181 (Date of registration; August 18, 2021).

### Eligibility criteria

Eligible participants included female patients with histologically confirmed HER2-positive primary breast cancer (defined as either Immunohistochemistry (IHC) 3+, or IHC 2+ with fluorescence in situ hybridization positivity) and no prior history of anti-HER2 therapy. Exclusion criteria included a history of metastatic or recurrent breast cancer, active concurrent malignancies, uncontrolled comorbidities, or corticosteroid/immunosuppressive therapy unrelated to the study protocol.

**Fig. 1:**
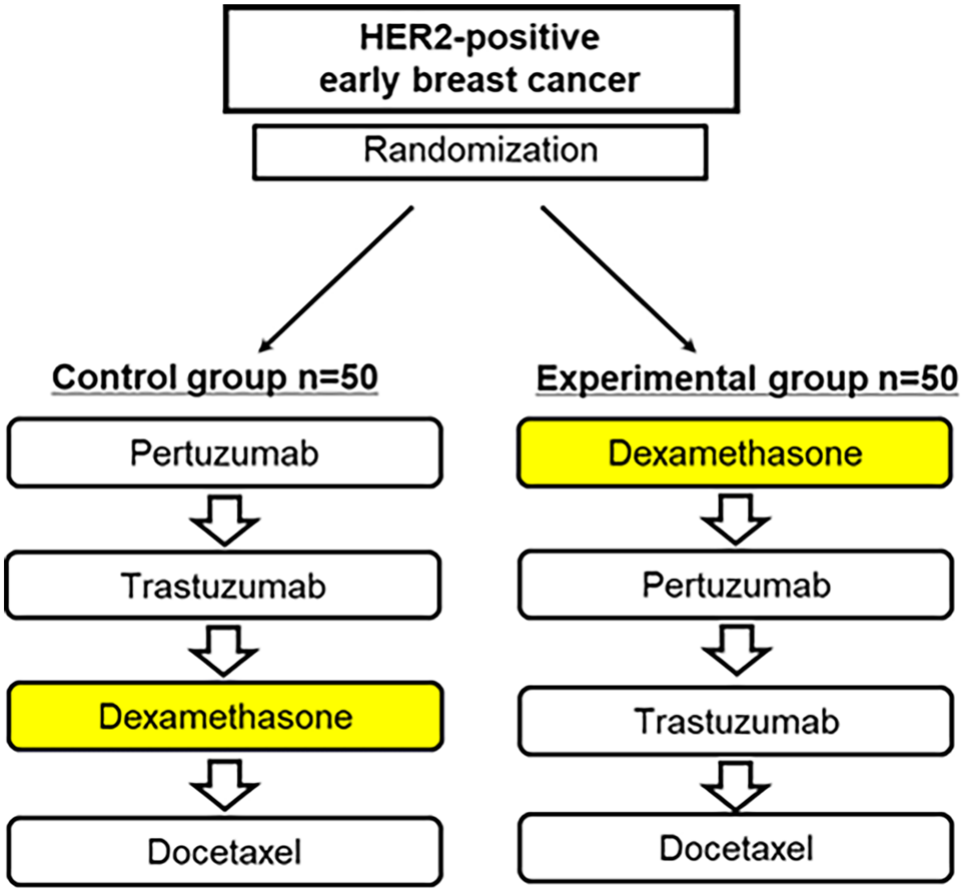
Study design and treatment allocation. Patients with HER2-positive early breast cancer were randomized (1:1) to either the experimental group (*n* = 50) or the control group (*n* = 50). In the control group, dexamethasone was administered following pertuzumab and trastuzumab but before docetaxel. In the treatment group, dexamethasone premedication was administered before HER2-targeted therapy (pertuzumab plus trastuzumab), followed by docetaxel

Treatment protocol (Fig. 1).

Patients in the control group received the standard premedication sequence:

1. Pertuzumab 840 mg IV over 60 min.
2. Trastuzumab 8 mg/kg IV over 90 min.
3. Dexamethasone 6.6 mg IV over 30 min.
4. Docetaxel 75 mg/m^2^ IV over 60 min.

Patients in the intervention group received dexamethasone 6.6 mg before HER2-targeted therapy. The sequence was as follows:

1. Dexamethasone 6.6 mg IV over 30 min.
2. Pertuzumab 840 mg IV over 60 min.
3. Trastuzumab 8 mg/kg IV over 90 min.
4. Docetaxel 75 mg/m^2^ IV over 60 min.

### Randomization

Patients were randomly assigned in a 1:1 ratio to either the experimental group or the control group. Randomization was performed centrally by the data center using a stratified block randomization method. The stratification factor was treatment setting (neoadjuvant vs. adjuvant chemotherapy). Eligible participants were enrolled by investigators at each participating site, and allocation was concealed via centralized randomization to ensure blinding of the sequence.

### Endpoints

The primary endpoint included the incidence of IRs during the first infusion cycle, as defined by the Common Terminology Criteria for Adverse Events version 5.0 (CTCAE v5.0). Secondary endpoints included the incidence of grade ≥ 3 IRs, overall adverse events, and IR rates during the second cycle. The premedication and dosing protocol, including the timing of dexamethasone administration, was identical for both the first and second cycles.

### Assessment of infusion reactions

IRs were assessed by the treating physicians or clinical staff at each participating site based on clinical observation and patient-reported symptoms. All assessments were conducted in accordance with CTCAE v5.0, as prespecified in the study protocol. Although the study was open-label and observers were not blinded to treatment allocation, standardized case report forms were used to document symptom type, onset timing, and severity grade. This protocol-driven approach was intended to ensure consistency in IR evaluation across all sites.

### Statistical analysis

The sample size was calculated assuming a 45% incidence of IRs in the control group and a reduction to 20% in the experimental group. This assumption was based on prior reports indicating up to 40% IR incidence with trastuzumab [11], as well as institutional retrospective data showing a 55.6% IR incidence without steroid premedication [12]. Assuming a one-sided alpha level of 5%, a power of at least 80%, and a 1:1 allocation ratio, the minimum required sample size was estimated to be 94 patients. To account for potential dropouts, the final target enrollment was set at 100 patients (50 per group).

The incidence of IRs was compared between groups using the *χ*^2^ test (chi-square test) or Fisher’s exact test, as appropriate. Confidence intervals for between-group differences were calculated using the Clopper–Pearson method. Continuous variables were expressed as mean ± standard deviation (SD), and categorical variables were reported as frequencies and percentages. Standardized mean differences (SMDs) were calculated to assess baseline balance between groups. SMD values below 0.1 were considered negligible, whereas those under 0.25 were considered acceptable. The overall distribution of SMDs was interpreted within the context of randomization. Because the baseline T-stage showed a standardized mean difference (SMD) of 0.327, an additional sensitivity analysis using logistic regression including treatment group and T-stage (T1 vs. ≥ T2) was conducted for the primary endpoint. Missing data were minimal and occurred completely at random. Therefore, all analyses were conducted using a complete-case approach without imputation. A *P* value of < 0.05 was considered statistically significant.

## Results

A total of 100 patients with HER2-positive early breast cancer were enrolled and randomized 1:1 to the experimental group (*n* = 50) or control group (*n* = 50). Baseline characteristics were well balanced between groups (Table 1). Most SMDs were less than 0.25, indicating good balance between groups. T-stage demonstrated a slightly higher SMD (0.327), suggesting minor variation between groups rather than substantial imbalance.

**Table 1:** Baseline patent characteristics

	Control group (*n* = 50)	Experimental group (*n* = 50)	*P* value	SMD
	*n* (%)	*n* (%)		
Age (Mean ± SD)	56.66 ± 10.85	58.84 ± 11.87	0.34	0.192
BMI (Mean ± SD)	22.16 ± 2.86	22.22 ± 3.83	0.921	0.020
Allergy			0.820	0.091
Yes	12 (24.0)	14 (28.0)		
No	38 (76.0)	36 (72.0)		
T-Stage			0.456	0.327
T1	18 (36.0)	24 (48.0)		
T2	25 (50.0)	23 (46.0)		
T3	3 (6.0)	1 (2.0)		
T4	4 (8.0)	2 (4.0)		
N-Stage			0.817	0.194
N0	29 (58.0)	31 (62.0)		
N1	15 (30.0)	14 (28.0)		
N2	1 (2.0)	2 (4.0)		
N3	5 (10.0)	3 (6.0)		
M0	50 (100.0)	50 (100.0)	NA	< 0.001
Stage			0.848	0.286
I	15 (30.0)	19 (38.0)		
IIA	15 (30.0)	16 (32.0)		
IIB	11 (22.0)	8 (16.0)		
IIIA	2 (4.0)	3 (6.0)		
IIIB	2 (4.0)	1 (2.0)		
IIIC	5 (10.0)	3 (6.0)		
Histological type			0.841	0.080
Invasive ductal carcinoma	28 (56.0)	26 (52.0)		
Others	22 (44.0)	24 (48.0)		
Histological grade			0.599	0.204
1	4 (8.0)	5 (10.0)		
2	24 (48.0)	19 (38.0)		
3	22 (44.0)	26 (52.0)		
ER			0.311	0.245
≥10	26 (52.0)	32 (64.0)		
< 10	24 (48.0)	18 (36.0)		
PgR			0.675	0.126
≥10	19 (38.0)	16 (32.0)		
< 10	31 (62.0)	34 (68.0)		
Ki-67			0.546	0.162
≥ 30	30 (60.0)	26 (52.0)		
< 30	20 (40.0)	24 (48.0)		
Treatment setting			1.000	0.045
Neoadjuvant	37 (74.0)	36 (72.0)		
Adjuvant	13 (26.0)	14 (28.0)		
Prior chemotherapy			0.365	0.083
AC or EC	24 (48.0)	26 (52.0)		
Dose dense AC or EC	9 (18.0)	8 (16.0)		
Others	1 (2.0)	1 (2.0)		
No	16 (32.0)	15 (30.0)		

**Fig. 2:**
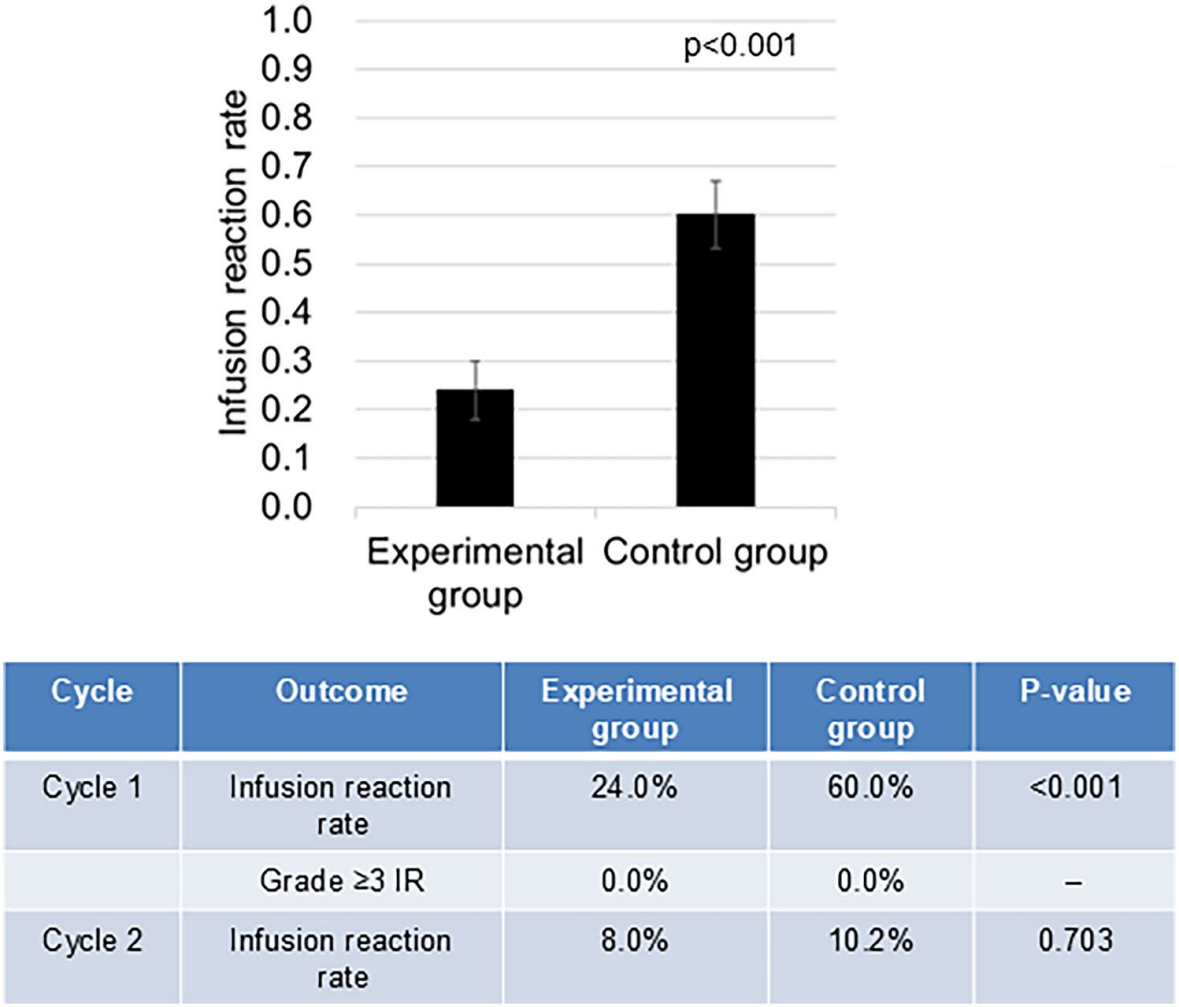
Incidence of infusion reactions (IRs) during the first and second treatment cycles. Incidence of IRs during cycle 1 (primary endpoint) was significantly lower in the experimental group than in the control group (24.0% vs 60.0%; *P* < 0.001). In cycle 2, IRs occurred in 8.0% and 10.2% of patients in the experimental and control groups, respectively; the difference was not statistically significant (*P* = 0.703)

Incidence of IRs during the first treatment cycle—the primary endpoint of this study—differed significantly between groups. IRs occurred in 24.0% of patients (12/50) in the experimental group, who received dexamethasone before HER2-targeted therapy, compared with 60.0% (30/50) in the control group, where dexamethasone was administered after HER2-targeted therapy. This represents an absolute risk reduction of 36.0% (95% confidence interval (CI) − 54.0% to − 18.0%). The difference was statistically significant based on both chi-square and Fisher’s exact tests (*P* < 0.001). These findings support the hypothesis that premedication with dexamethasone before HER2-targeted therapy significantly reduces the incidence of IRs during the first cycle of treatment (Fig. 2).

As a secondary analysis, the incidence of IRs during the second cycle was evaluated. IRs occurred in 9 (9.1%) of 99 evaluable patients. The incidence was slightly lower in the experimental group (8.0%, 4/50) than in the control group (10.2%, 5/49), but the difference was not statistically significant (chi-square test, *P* = 0.703; Fisher’s exact test, *P* = 0.741; Fig. 2). Notably, no grade ≥ 3 IRs were reported in either group during the first cycle, precluding statistical comparison for this endpoint (Fig. 2).

Although several specific infusion-related adverse events (including chills, flushing, headache, nausea, and vomiting) were more frequent in the control group versus the experimental group, the overall incidence of treatment-related adverse events (TRAEs) was similar between groups. TRAEs occurred in 98.0% of patients in the experimental group and 100.0% in the control group, with no statistically significant difference between groups (chi-square and Fisher’s exact test, *P* > 0.999). The results suggest that the modified premedication sequence effectively reduced IR-related events without significantly altering the overall treatment-related toxicity profile (Table 2).

**Table 2:** Comparison of treatment-related adverse events between experimental and control groups

TRAE	Control arm (*n* = 50)			Experimental arm (*n* = 50)			Any grade
	Any Grade (%)	Grade 3	IR	Any Grade (%)	Grade 3	IR	*P* value
Any TRAE	49 (100)	2	30	49 (98)	10	12	1.000
Nausea	18 (36)	0	0	18 (36)	2	1	1.000
Flushing	22 (44)	0	13	14 (28)	0	6	0.097
Head ache	14 (28)	0	8	14 (28)	0	5	1.000
Chills	25 (50)	0	24	10 (20)	1	6	0.002
Vomiting	14 (28)	0	12	2 (4)	0	1	0.001
Fatigue	41 (82)	0	0	42 (84)	2	0	1.000
Anorexia	31 (62)	2	0	40 (80)	6	0	0.077
Mood changes	8 (16)	0	0	17 (34)	0	0	0.063
Diarrhea	18 (36)	0	0	16 (32)	1	0	0.675
Pruritus	21 (42)	0	0	15 (30)	0	0	0.214
Edema	18 (36)	0	0	12 (24)	0	0	0.194
Stomatitis	7 (14)	0	0	6 (12)	1	0	0.774
Rash	13 (26)	0	0	6 (12)	0	0	0.078
Pharyngolaryngeal hypoesthesia	3 (6)	0	0	5 (10)	0	0	0.715
Arthralgia	5 (10)	0	0	3 (6)	0	0	0.487
Constipation	3 (6)	0	0	3 (6)	0	0	1.000
Dysgeusia	0	0	0	0 (0)	0	0	1.000

**Fig. 3:**
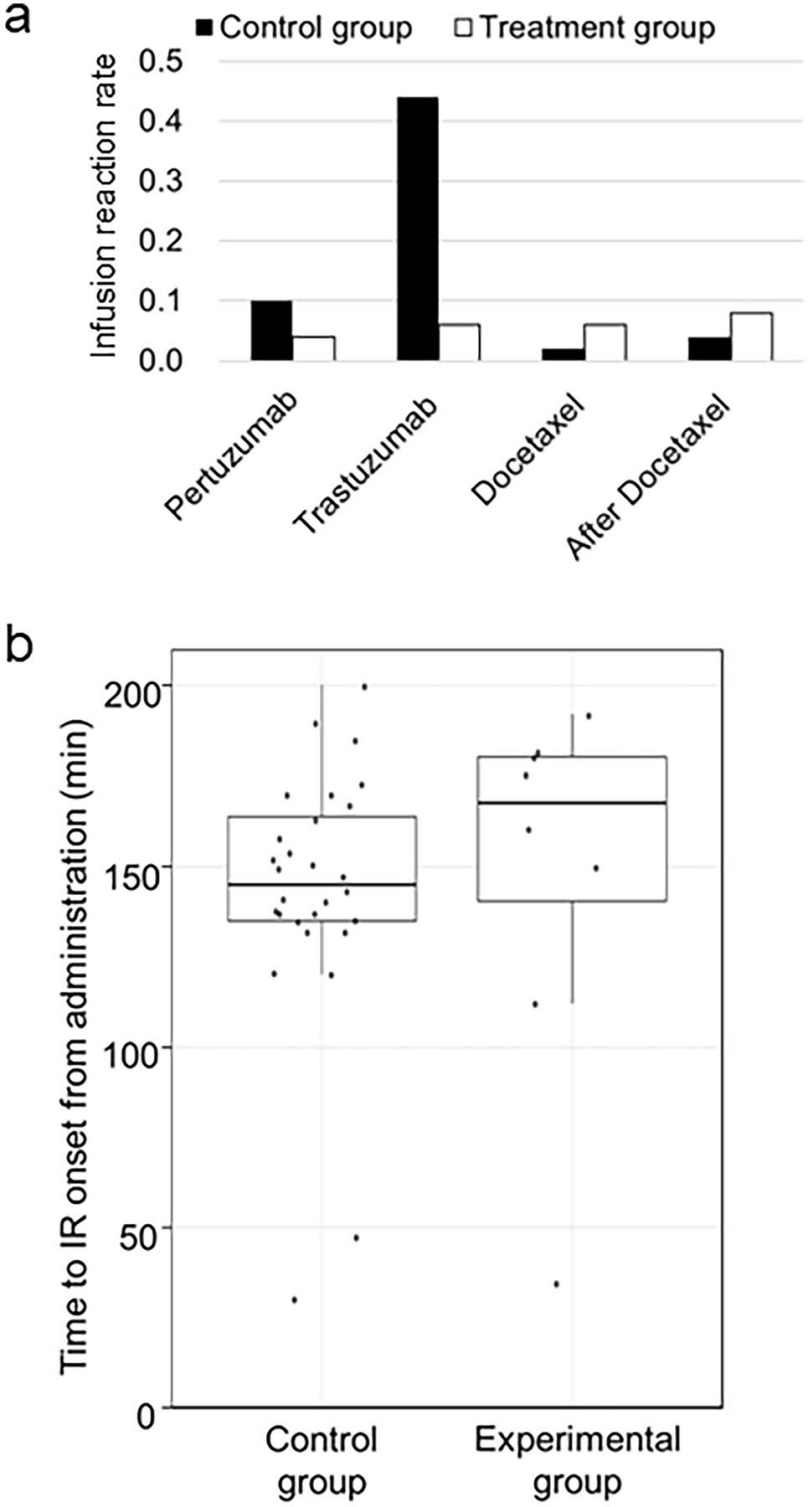
Timing and distribution of infusion reactions during the first treatment cycle. **a** Distribution of infusion reactions (IRs) by treatment phase. IRs were classified into four distinct phases: Pertuzumab: from the start of pertuzumab infusion to the start of trastuzumab infusion, Trastuzumab: from the start of trastuzumab infusion to the start of docetaxel infusion, Docetaxel: during docetaxel administration, After Docetaxel: following docetaxel treatment. The number of IR events within each time window is shown for both treatment groups. **b** Time plot of IR onset during the first treatment cycle. Each IR event is plotted along the infusion timeline, from the start of the first drug to the end of the last drug administered. In the control group, several IRs occurred before dexamethasone administration, particularly during pertuzumab and trastuzumab infusions. This temporal pattern suggests that delayed steroid premedication may not adequately prevent early-onset IRs. Conversely, the experimental group—which received premedication with dexamethasone before HER2-targeted therapy experienced a lower incidence of IRs during the initial infusion period

A detailed analysis of IR onset timing was conducted to further assess the impact of dexamethasone administration timing. IRs during the first cycle were categorized into four distinct phases: (1) from the start of pertuzumab to the start of trastuzumab, (2) from the start of trastuzumab to the start of docetaxel, (3) during docetaxel administration, and (4) after completion of all infusions. As shown in Fig. 3a, IRs occurred more frequently in the control group during the early phases, particularly with HER2-targeted agent infusion before dexamethasone administration. In contrast, the experimental group showed a lower incidence of IRs during the early phases, suggesting that earlier dexamethasone administration may protect against early-onset IRs. Figure 3b shows the temporal distribution of IR events in individual patients, confirming that a substantial proportion of IRs in the control group occurred before dexamethasone administration.

Finally, we evaluated the potential association between baseline laboratory parameters and the risk of IRs. Table 3 compares pre-treatment white blood cell count, neutrophil-to-lymphocyte ratio (NLR), liver function tests [aspartate aminotransferase (AST), alanine aminotransferase (ALT)], and renal function (serum creatinine) between the control and experimental groups with different timing of dexamethasone administration. No statistically significant differences were observed between the groups. These findings suggest that baseline laboratory values were not predictive of IR development, whereas the timing of dexamethasone administration played a critical role in modulating IR risk.

**Table 3:** Association between baseline laboratory values and incidence of infusion reactions

	Control group (*n* = 50)	Experimental group (*n* = 50)	P value	SMD
	Mean (SD)	Mean (SD)		
White blood cell	6032.20 (3578.66)	6239.00 (3342.19)	0.766	0.060
Neutrophil	4319.70 (3158.88)	4509.24 (3251.35)	0.768	0.059
Lymphocyte	1135.08 (501.69)	1142.44 (956.25)	0.962	0.010
Neutrophil-to-lymphocyte ratio	4.43 (3.80)	5.12 (4.57)	0.411	0.165
Platelet	28.87 (7.94)	38.26 (45.96)	0.158	0.285
Total bilirubin	0.47 (0.20)	0.50 (0.21)	0.546	0.121
Creatinine	0.60 (0.10)	0.61 (0.13)	0.610	0.103
Alanine aminotransferase	19.68 (14.52)	20.36 (12.25)	0.801	0.051
Aspartate aminotransferase	19.86 (8.26)	21.36 (8.49)	0.373	0.179
Alkaline phosphatase	77.62 (26.23)	82.40 (33.59)	0.430	0.159

## Discussion

This randomized clinical trial demonstrates that modifying the sequence of dexamethasone administration significantly reduces the incidence of IRs in patients receiving HER2-targeted therapy for early-stage HER2-positive breast cancer. Specifically, administering dexamethasone before pertuzumab and trastuzumab significantly reduced the rate of IRs during cycle 1, compared to the standard sequence where dexamethasone is administered only before docetaxel. These findings support the hypothesis that earlier corticosteroid premedication confers protective benefits during biologic agent infusions, extending beyond its established role in cytotoxic chemotherapy.

There was a statistically significant and clinically meaningful 36% absolute reduction in IR incidence, from 60.0% to 24.0% (*P* < 0.05, 95% CI excludes zero). This result is consistent with previous retrospective studies indicating that early corticosteroid administration may reduce IRs associated with monoclonal antibody therapies [11–15]. However, this study represents the first prospective, randomized evidence supporting dual HER2 blockade using trastuzumab and pertuzumab. Notably, no grade ≥ 3 IRs were observed in either group. However, while most IRs were low-grade, they remained common and could disrupt treatment continuity and patient experience. This may reflect limited power to detect rare severe events. The modified premedication sequence may primarily reduce mild-to-moderate IRs, which still affect patient comfort and adherence. Larger studies are needed to assess its impact on severe IRs. Similarly, the lack of a significant difference in cycle 2 may be due to immune tolerance developed after initial exposure during cycle 1, leading to an overall lower baseline risk in subsequent cycles. Nevertheless, the possibility of insufficient power to detect small intergroup differences cannot be excluded. These findings underscore that the benefit of early dexamethasone administration is most pronounced during the first cycle.

Importantly, the benefit of the modified dexamethasone sequence was primarily observed during the first treatment cycle, when the immune system typically shows the strongest response to newly introduced monoclonal antibodies. In the second cycle, IR rates were low and comparable between treatment groups, consistent with the expected development of immune tolerance following initial exposure. This temporal pattern underscores the importance of optimizing premedication strategies, particularly during the initial administration of HER2-targeted agents.

Although the modified premedication sequence significantly reduced IRs—particularly chills, flushing, and vomiting—the overall incidence of TRAEs remained high and comparable between groups. This suggests that while the timing of dexamethasone administration effectively mitigates acute immune-related reactions commonly observed with monoclonal antibodies, it does not influence the broader toxicity profile of chemotherapy or HER2 blockade. These findings are reassuring from a safety perspective and offer practical insight into which IR components may be most responsive to premedication optimization.

A detailed time-course analysis showed that IRs in the control group occurred primarily during or immediately after pertuzumab and trastuzumab infusion—before dexamethasone administration. In contrast, IRs in the treatment group were less frequent and more evenly distributed across subsequent time points, suggesting that early corticosteroid administration effectively prevents acute IRs to HER2-targeted therapy. These findings have practical implications, suggesting that modifying the infusion order may serve as a simple yet effective strategy to improve patient comfort and treatment tolerability.

Although previous guidelines, including those from the European Society for Medical Oncology (ESMO), do not routinely recommend corticosteroid premedication for trastuzumab [13, 16], our findings suggest that a modified premedication regimen may provide significant clinical benefits.

These recommendations primarily address whether corticosteroids should be used for single-agent HER2-targeted therapy, but do not consider the timing of administration in combination regimens that include cytotoxic agents. Importantly, our study does not aim to reassess the necessity of steroids for trastuzumab monotherapy, but rather emphasizes the clinical relevance of modifying the sequence of administration when corticosteroids are already indicated—for example, for docetaxel—to enhance protection against early-onset IRs. Furthermore, our findings are consistent with prior studies of monoclonal antibodies such as cetuximab and rituximab, for which corticosteroid premedication has been shown to reduce hypersensitivity reactions [3, 17]. This trial extends those insights to HER2-targeted regimens, highlighting the critical importance of steroid timing in mitigating IRs.

Furthermore, the implications of this study may extend beyond HER2-positive breast cancer. IRs are a common adverse effect of monoclonal antibody-based therapies, which are widely used in both oncologic and non-oncologic diseases [3]. Agents, such as immune checkpoint inhibitors (e.g., pembrolizumab, nivolumab) and anti-cluster of differentiation 20 (anti-CD20) monoclonal antibodies (e.g., rituximab) are associated with high rates of IRs [18, 19]. For these therapies, optimizing premedication strategies—particularly the timing of corticosteroid administration—may improve tolerability, minimize treatment interruptions, and improve patient adherence.

In this study, no patients received oral dexamethasone prior to treatment. All patients were administered intravenous dexamethasone (6.6 mg) on the day of infusion as per protocol, and oral dexamethasone was prescribed for two days after treatment to prevent docetaxel-induced edema. Antipyretic or analgesic agents such as acetaminophen and NSAIDs were also not included in the premedication protocol. In Japan, prophylactic use of these agents for infusion reaction prevention in this context is considered off-label, and their use may vary across institutions. While these agents may be used at the discretion of clinicians, their potential role—either in combination with or as an alternative to corticosteroid premedication—was beyond the scope of this trial.

Therefore, the observed reduction in infusion reactions in our study can be attributed to the timing of intravenous dexamethasone administration on the day of treatment. Future studies may further investigate these adjunctive strategies, although their necessity should be weighed against current clinical practices and the established efficacy of steroid-based approaches.

No association was found between baseline laboratory parameters (white blood cell counts, NLR, liver enzymes, and renal function) and the development of IRs. This finding suggests that routine blood tests may have limited clinical utility in predicting IR risk. Instead, focusing on modifiable factors—particularly premedication protocols—may provide a more effective preventive strategy.

This study has several limitations. First, while the sample size was sufficient to detect differences in IR rates, it may not have been adequately powered to detect differences in less frequent adverse events or within specific patient subgroups. Second, the study population comprised exclusively patients with early-stage disease receiving standard perioperative therapy; consequently, the findings may not be generalizable to metastatic settings or patients receiving alternative HER2-targeted treatments. However, this curative-intent setting represents a critical context in which treatment tolerability is essential. This trial therefore provides definitive guidance for optimizing premedication protocols in early-stage HER2-positive breast cancer, while highlighting the need for further research in metastatic disease. Third, the assessment of IRs relied solely on clinical symptoms and timing, without incorporating biomarkers or mechanistic assays to characterize the underlying immune response. In addition, a numerically higher incidence of grade ≥ 3 adverse events was observed in the experimental group. However, this analysis was based on the secondary endpoints that were not powered for formal statistical comparison, and multiple safety outcomes were explored without adjustment for multiplicity. Therefore, this finding should be interpreted with caution, as it may reflect random variation rather than a true difference in toxicity between groups. Finally, while there were no missing data for predefined endpoints, some minor or subjective symptoms may not have been consistently reported across sites. Despite the use of standardized CRFs, a minimal risk of underreporting remains, which is an inherent limitation in multicenter data collection.

Regardless of these limitations, the study demonstrates that optimizing dexamethasone timing represents a practical, cost-effective strategy for reducing IRs in patients receiving trastuzumab and pertuzumab therapy. Based on these findings, modifying institutional premedication protocols to include dexamethasone prior to HER2-targeted therapy—particularly during the first treatment cycle—may be feasible. This approach’s simplicity and potential applicability to other monoclonal antibody therapies highlights its relevance across diverse clinical settings.

## Conclusion

This randomized clinical trial provides the first prospective evidence that modifying the sequence of dexamethasone administration significantly reduces infusion reactions associated with dual HER2-targeted therapy in early-stage breast cancer. Administering dexamethasone before (rather than after) trastuzumab and pertuzumab significantly reduced the incidence of IRs without increasing other treatment-related toxicities. These findings suggest that a simple adjustment to the premedication protocol could improve patient safety and comfort during HER2-targeted therapy. This approach may also be applicable to other monoclonal antibody-based therapies, warranting a re-evaluation of standard premedication protocols in oncology and other therapeutic areas.

## Data Availability

The datasets generated and/or analyzed during the current study are available from the corresponding author upon reasonable request.

## Abbreviations

ESMO: European Society for Medical Oncology
IR: Infusion reaction
NLR: Neutrophil-to-lymphocyte ratio
SD: Standard deviation
TRAE: Treatment-related adverse events
